# The Dresden Protocol for Multidimensional Walking Assessment (DMWA) in Clinical Practice

**DOI:** 10.3389/fnins.2020.582046

**Published:** 2020-10-26

**Authors:** Katrin Trentzsch, Marie Luise Weidemann, Charlotte Torp, Hernan Inojosa, Maria Scholz, Rocco Haase, Dirk Schriefer, Katja Akgün, Tjalf Ziemssen

**Affiliations:** Department of Neurology, MS Center Dresden, Center of Clinical Neuroscience, Neurological Clinic, University Hospital Carl Gustav Carus, TU Dresden, Dresden, Germany

**Keywords:** multiple sclerosis, gait analysis, mobility, phenotyping, wearable sensors

## Abstract

Walking impairments represent one of the most debilitating symptom areas for people with multiple sclerosis (MS). It is important to detect even slightest walking impairments in order to start and optimize necessary interventions in time to counteract further progression of the disability. For this reason, a regular monitoring through gait analysis is highly necessary. At advanced stages of MS with significant walking impairment, this assessment is also necessary to optimize symptomatic treatment, choose the most suitable walking aid and plan individualized rehabilitation. In clinical practice, walking impairment is only assessed at higher levels of the disease using e.g., the Expanded Disability Status Scale (EDSS). In contrast to the EDSS, standardized functional tests such as walking speed, walking endurance and balance as well as walking quality and gait-related patient-reported outcomes allow a more holistic and sensitive assessment of walking impairment. In recent years, the MS Center Dresden has established a standardized monitoring procedure for the routine multidimensional assessment of gait and balance disorders. In the following protocol, we present the techniques and procedures for the analysis of gait and balance of people with MS at the MS Center Dresden. Patients are assessed with a multidimensional gait analysis at least once a year. This enables long-term monitoring of walking impairment, which allows early active intervention regarding further progression of disease and improves the current standard clinical practice.

## Introduction

Multiple sclerosis (MS) is a common chronic inflammatory disease of the central nervous system that is clinically characterized by a distinct heterogeneity (Disanto et al., 2011; Bassi et al., 2017). This is caused – amongst others – by disseminated inflammatory lesions in the central nervous system that initially lead to disorders of single functional systems (FS), in the course of the disease of multiple FS with a wide variety of neurological deficits (Lindner et al., 2018). Lord Kelvin made the important observation that “what you cannot measure, you cannot improve.” Applied to the current, increasingly complex management of MS, this means that the individual multidimensional disease characteristics of people with MS (pwMS) should be made as quantifiable as possible in order to enable phenotyping of the individual disease characteristics and longitudinal monitoring of these parameters (Bassi et al., 2017). In the beginning of the course of the disease, it is still possible to functionally compensate for the damage caused by the demyelinating lesions through cerebral restructuring and compensation processes (Ziemssen et al., 2015, 2016a). However, over the course of the disease, these mechanisms become increasingly exhausted, resulting in incomplete remission of symptoms regression or clinical progression (Inojosa et al., 2019).

In the context of phenotyping MS, different dimensions and perspectives must be distinguished (Ziemssen et al., 2016b). Several neurological-clinical domains should be part of the quantitative assessment of individual neurological FS (e.g., cognition, walking) as eg. imaging (magnetic resonance imaging, ocular coherence tomography) and electrophysiological procedures, patient-reported outcomes (PRO), new molecular (e.g., neurofilament light chain) and digital biomarkers (D’Amico et al., 2019; Ziemssen et al., 2019; Inojosa et al., 2020a). The ability to walk is an important prerequisite to participate autonomously in daily life. Accordingly, walking impairment (WI) leads to reduced mobility and autonomy and thus to a lower quality of life. About 85% of pwMS reported WI (Comber et al., 2017), and about 70% of pwMS considered WI as the major issue of their disease (LaRocca, 2011; Heesen et al., 2018). In almost half of the cases (48%), WI resulted in a need of a walking aid 15 years after onset of symptoms (Kister et al., 2013). A various number of pathophysiological mechanisms in different neurological systems contribute to WI, e.g., partial or complete paralysis and spasticity play a predominant role, but also the cerebellar, balance, sensory, fatigue-related, cognitive and even visual dysfunction (Goldman et al., 2008; Shanahan et al., 2018). Consequently, it is important to identify subtle signs of WI as early as possible to adjust the disease-modifying treatment and prevent further progression (Ziemssen and Thomas, 2017). Furthermore, more attention needs to be paid to balance disorders as they may be indicators for falls (Brandstadter et al., 2020; Inojosa et al., 2020b). A regular monitoring of individual WI is necessary to assess this relevant FS (Hobart et al., 2019). At advanced stages of MS, the assessment of patients with significant WI is necessary to optimize symptomatic treatment (e.g., with fampridine), choose a suitable aid and develop individualized rehabilitation concepts (Rodriguez-Leal et al., 2018; Playford, 2019).

In clinical practice, the Expanded Disability Status Scale (EDSS) and the Multiple Sclerosis Functional Composite (MSFC) are the most established clinical scales that include WI. The EDSS assesses the maximum possible walking distance up to 500 m and the MSFC measures the walking speed for a 7.62 m distance using the timed 25-foot walking (T25FW) test (Kurtzke and Hutchinson, 1983; Cutter et al., 1999; Inojosa et al., 2020a). However, these rather rough and one-dimensional estimates lack sufficient sensitivity for initially very subtle WI (Shanahan et al., 2018). Consequently, early abnormalities or small changes in long-term monitoring may not be detected. Here, the integration of new technologies and standardized non-human-dependent measures appear as promising tools.

A multidimensional gait analysis that not only evaluates the walking distance but also the quality of gait can provide important and practically relevant data as it has been reported in various studies where, for example, one single test, such as the maximal walking distance and walking speed, does not fully describe WI in pwMS. As significant abnormalities are already present even in mildly impaired pwMS, the analysis of the trunk fluctuation range should be integral part of multidimensional gait analysis of pwMS (Martin et al., 2006; Pau et al., 2017). The combination of static and dynamic balance and gait tests offers a complete evaluation of the gait in pwMS. In this methodological paper of our assessment protocol, we discuss current possibilities for walking assessment and their suitability for the specialized clinical practice including the required infrastructure and present the standard of care at the Multiple Sclerosis Center (MSC) of the University Hospital Carl Gustav Carus (Dresden, Germany). Applying these methods, we aim to collect high-quality clinically relevant data for the characterization of pwMS in a real world setting and thus understand the different alterations in gait and balance in these patients.

## Walking Assessment of People With MS in Research

The attention in research on mobility in pwMS has shifted to advanced technologies of movement analysis, promising higher sensitivity for early stages of impairment than standardized clinical assessments (Shanahan et al., 2018). However, a more advanced and objective gait analysis goes beyond a chronometer or a plain observation of the patient. Research on mobility therefore needs a complex infrastructure to generate quality data. Hereinafter, we give an overview of the assessment technology and describe associated outcomes that have been investigated in MS (Table 1).

**TABLE 1:** Assessment technologies for advanced gait analysis.

Assessment technology	Method	Outcomes*	Device^†^(Manufacturer)
Video-based	a) marker based b) marker-free	a) & b) joint range of motion	a) Vicon (Vicon Motion Systems Ltd); Miqus Hybrid (Qualisys AB) b) Kinect (Microsoft); Miqus Hybrid (Qualisys AB)
Sensor floor plates	a) instrumented walkway b) force platform c) balance boards	a) spatiotemporal measures b) ground reaction force pattern c) ground reaction force pattern	a) GAITRite (CIR Systems) b) ProKin (Tecnobody); 3D Force Plate (Kistler Instruments AG) c) Wii Balance Board (Nintendo)
Wearable sensors	a) research-oriented^‡^ b) consumer-driven^∫^	a) & b) spatiotemporal measures joint range of motion	a) mobility lab (APDM), XActiGraph GT9X Link (ActiGraph); GENEActiv Original (Activinsights) b) Fitbit Charge 4 (fitbit), vívosport^®^ (Garmin), Xiaomi Mi Band 4 (Xiaomi)

*^∗^Selection of key Outcomes; ^†^examples; ^‡^devices developed primarily for research purposes: no direct patient feedback, no modifying of movement behavior through e.g. motivation, raw data output; ^∫^devices developed primarily for consumer requirements: direct feedback of movement behavior on device display, no direct access to raw data.*

### Video-Based Analysis Systems

Video-based systems capture a joint range of motion (kinematics) operating with or without markers that are placed on anatomic landmarks. Marker-based systems show high accuracy and reproducibility, whereas marker-free systems are less accurate but more user-friendly. Especially marker-based systems involve extensive technological and human resources as well as a longer preparation time reducing their practicability in clinical use (Shanahan et al., 2018). Although most of the trials combined video analysis techniques with force plates or electromyography (EMG), which assess spatiotemporal measurements, and connect kinematics with ground reaction forces or EMG results (Benedetti et al., 1999; Martin et al., 2006; Galea et al., 2017), some of the camera systems can generate spatiotemporal parameters independently (Behrens et al., 2014; Liparoti et al., 2019). Benedetti et al. found that pwMS with EDSS 0-2 had increased flexion in hip, knee and ankle plantarflexion during initial contact, and reduced extension of hip and knee at the toe off phase. The authors suggested that the differences in kinematics are derived by compensation of ankle stiffness and general loss of fine motor skills (Benedetti et al., 1999). Another study comparing pwMS without pyramidal signs, pwMS with mild pyramidal signs and healthy controls confirmed the findings of increased ankle plantarflexion during the initial contact. Significant differences were found between both MS groups and controls. In contrast to Benedetti, this study did not find differences in knee kinematics (Martin et al., 2006). By examining kinematic changes of relapsing-remitting MS (RRMS)-patients over a 12-month period, Galea et al. found differences regarding the ankle angle in the group of patients without relapse within the year. However, kinematics did not differ in gait impairment, functional reach and gait speed. Further, the EDSS did not reflect the progress of impairment (Galea et al., 2017). The study of Liparoti et al. showed a decrease in velocity and stability parameters in pwMS with minimal impairment during single task as well as dual task condition confirming previous observations of those spatiotemporal measurements. In regard of kinematics, they found an increased ankle dorsiflexion in the phase of single support, which they considered to be the earliest change in kinematics associated with velocity and stability changes (Liparoti et al., 2019).

### Sensor Floor Plates

Floor integrated sensors comprise instrumented walkways, force plates and, similar to the latter, portable balance boards (Shanahan et al., 2018). Instrumented walkways measure spatiotemporal gait parameters via pressure sensors, integrated in an electronic portable carpet (Cameron and Wagner, 2011). The GAITRite from CIR Systems is the most widely used walkway that has been the subject of numerous studies in MS (Sosnoff et al., 2012; Psarakis et al., 2017). It has been shown to be valid and reliable regarding spatiotemporal parameters (Bilney et al., 2003; Menz et al., 2004). Force platforms operate with strain gauge or piezoelectric sensors and can be integrated in a walkway or a treadmill to capture multiple gait cycles. In gait analysis, they provide a curve of ground reaction force. In a balance assessment, a center of pressure diagram is recorded (Shanahan et al., 2018). Both gait and balance parameters have shown high test-retest reliability (Goldie et al., 1989; Pinsault and Vuillerme, 2009). Using a treadmill integrated system, Kalron et al. found that minimally impaired pwMS chose a slower jogging tempo, with wider steps and higher double support time, which might be attributed to a safety strategy (Kalron et al., 2013). In balance assessment, a recent study suggested a high sensitivity of the range of the sway area from the patient’s center of gravity as well as of the average speed, to uncover early balance deficits in pwMS not detectable for the physician (Inojosa et al., 2020b). Regarding a risk-of-fall screening, balance outcomes measured by force platforms showed better discriminative properties compared to the Berg Balance Score (Prosperini et al., 2013). In the last years, portable balance boards have been investigated as an alternative to force platforms in balance assessment due to simple handling and low cost. The Nintendo Wii balance board was considered as a valid and reliable alternative in balance assessment of pwMS (Clark et al., 2018; Sun et al., 2018).

### Wearable Sensor Systems

Inertial sensors for mobility assessment can be used in the clinical settings as well as at home (Weidemann et al., 2019a). A recent review provides an oversight of research in MS using inertial sensors and presented 17 studies on this subject (Frechette et al., 2019). Applied in a clinical gait lab, these sensors have shown good psychometric properties (Sun et al., 2018). They were able to discriminate pwMS from healthy controls with a higher sensitivity than stopwatch tests (Spain et al., 2012). Hilfiker et al. demonstrated a higher responsiveness of sensor derived parameters to changes within a three-week rehabilitation program compared to other gait performance tests (Hilfiker et al., 2013). However, in a study of 18-month observation, no worsening of parameters could be found, suggesting influence of daily fluctuations of symptoms (Spain et al., 2014).

Home-based assessment allows an evaluation of activity and especially of participation according to the classification of the World Health Organization. Moreover, they promise the advantage of the results not being affected by daily fluctuation as repeated measures can be easily performed (Petraglia et al., 2019; Weidemann et al., 2019a). In MS, different sensors have been tested in the home environment, with Actigraph (ActiGraph Corp.) being most often studied. Predominantly, outcome measures were “activity count” and “step count” (Giggins et al., 2017). While in some sensors the validity and reliability in the MS cohort is limited (Kayes et al., 2009; Motl et al., 2012), two Actigraph products and the RT3 achieved good results (Hale et al., 2008; Learmonth et al., 2013; Motl et al., 2014). For the Actigraph GT3X, Learmonth et al. found minimal detectable changes (MDC) of 47% (without walking aid) and 67% (with walking aid) regarding counts per day as well as of 40% and 65% regarding steps per day. In comparison, the T25FW (12%/36%) and the Six-Minute Walk (11%/39%) provided smaller MDCs (Learmonth et al., 2013). With growing interest in the research on consumer devices, some studies have examined FitBit devices in pwMS. Block et al. tracked physical activity of 95 pwMS continuously over one year finding a high retention rate, correlations with established disability outcome measures and indications that the step count is more sensitive for changes. Finally, pwMS with a low baseline daily step count were found to be at higher risk of increased impairment after 1 year (Block et al., 2019).

Unfortunately, most of the research approaches have not been transferred to clinical practice so far. The complex infrastructure needed for this approach is often very expensive while testing and evaluation are time consuming and need well-trained staff and sufficient space. The complex task and the high amount of data are difficult to interpret because standardization is yet difficult at this stage. But there are initial steps to transfer these research approaches and technology into more user-friendly systems and to increase their applicability in clinical practice (Domínguez et al., 2020).

## Walking Assessment of People With MS in Clinical Practice

In clinical practice, a test battery of different components for walking assessment has to be developed, as one test alone is not able to describe the complex walking functions. In this context, walking assessment is more about individual monitoring to detect significant changes in walking as a biomarker for WI. In contrast to research in a controlled setting with the aim of deriving generally valid estimates that are as precise as possible, the results of a multidimensional gait analysis in the clinical practice have to be evaluated on an individual basis and interpreted together with the treating physician. Recently, Decavel et al. presented a multimodal assessment of gait that was recommended for clinical evaluation and research considering all measurement methods with good reproducibility. This included walking with and without dual task condition using the GAITRite system, the T25FW, the 6-minute walk test (6MWT) and further tests taking up to two hours per patient (Decavel and Sagawa, 2019). This is a good example of the fact that gait analysis in clinical practice is always a trade-off between different requirements, including the feasibility in terms of time and resources. Consequently, a requirement profile adapted to the respective facility must be created that takes these key points into account:

• What methods are recommended based on the state of research, what is required by authorities? Gait analysis is a process of interdisciplinary exchange that must be adapted to the advancing technological possibilities.
• What level of detail of the assessment is required? A screening approach requires different instruments than a targeted monitoring of a symptomatic therapy.
• What human and institutional resources can be allocated? There are great differences in resources between individual treatment centers.

Since 2015, in the course of setting up our gait laboratory, we have been dealing with the question of which methods are suitable for a specialized center and for normal neurological practices. Therefore, we gathered an expert group consisting of neurologists, physiotherapists, psychologists and MS nurses at the MSC to define what methods are necessary to perform a feasible but also detailed gait analysis in a center with over 1,500 patients per year. Our aim was to comprehensively record the relevant dimensions of complex walking function in pwMS, which have not been sufficiently considered so far. Due to limited time and human resources, such a protocol of a test battery had to be straightforward and easy to apply to patients in clinical practice. We defined the following key points for protocol generation:

• Consider the published state of research and learn from other centers.
• Create a feasible program that can be used daily by the vast majority of patients and staff.
• Establish a protocol, which enables regular long-term monitoring of all those treated at least once a year.
• Consider different degrees of detail in multidimensional gait analysis from screening to maximum assessment to make the approach scalable to deal with different available resources.

## The Dresden Protocol for Multidimensional Walking Assessment

**FIGURE 1:**
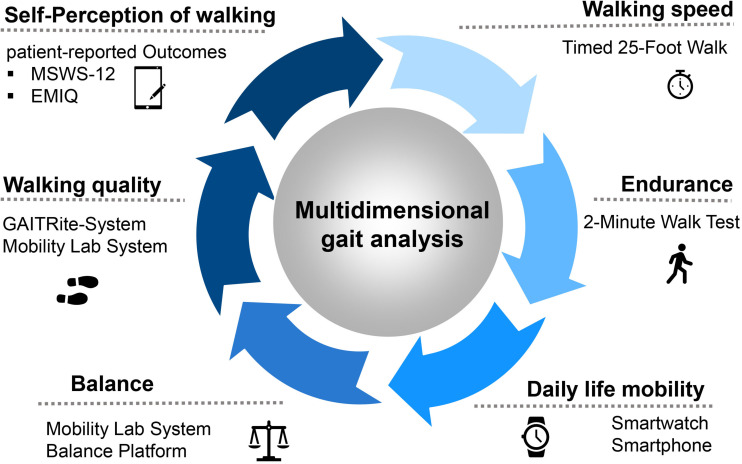
The protocol for the multidimensional walking assessment in clinical practice at the MS Center Dresden. Abbreviations: MSWS-12, Multiple Sclerosis Walking Scale; EMIQ, Early Mobility Impairment Questionnaire.

Our focus in multidimensional gait analysis is on the key components of the function of lower extremities. The Dresden protocol for multidimensional walking assessment (DMWA) includes the testing of **walking speed** (T25FW), **walking endurance** (2-minute walk test (2-MWT)), a **balance test** as well as the measurement of **walking quality** on a sensor-based walking mat (Figure 1). In addition, **self-perception of walking by the patient** is recorded applying the Twelve Item MS Walking Scale (MSWS-12) and Early Mobility Impairment questionnaire (EMIQ).

Regarding gait quality, Vienne et al. classify seven parameters for this multidimensional construct in the following areas: springiness, sturdiness, smoothness, stability, steadiness, symmetry and synchronization. Launching this index of gait quality, Vienne et al. argue that gait speed should be classified as a performance parameter rather than gait quality, especially the walking speed assessed in tests on maximum walking speed (e.g., T25FW). In the following, we do not assign the walking speed derived from the T25FW to gait quality but consider walking speed at a self-selected speed as a domain of walking quality because of it is indisputable influence on the other quality domains (Vienne et al., 2017).

Yearly multidimensional walking assessments of our pwMS are planned. Depending on the individual course of the disease (e.g., by changes in symptomatic therapy or acute relapses), more frequent assessments may be performed. In this order of ideas, patients with acute motor relapses and relevant walking impairment obtain an additional test before and after cortison administration to objectify new symptoms and response to therapy. Similarly, in case of starting a new symptomatic drug like e.g., antispastics or fampridine (Vienne et al., 2017) our patients get an additional walking assessment to objectify results and facilitate the decision of our physicians regarding continuation or stopping in case of non-responders. Since gait impairment is the major symptom of patients with primary progressive Multiple Sclerosis (PPMS), these patients perform a more continuous gait assessment every six months.

### Infrastructure

For the DMWA, we use the following infrastructure: the gait laboratory includes various camera- and sensor-based measuring systems. For example, pressure and movement sensors, plus additional force plates to record forces acting on the body in detail. In addition to the examinations in the gait lab, the evaluation of the 2-MWT is carried out on a corridor with a length of approximately 40 m. Motion capture systems were deliberately not considered for routine monitoring because we prefer techniques that are more practical for clinical use in the terms of time and personnel resources and our high number of patients analyzed daily.

The GAITRite System (CIR-Systems, Franklin, NJ, United States) is a single-layer walkway with pressure sensors, which allows the evaluation of various temporal and spatial gait parameters by recording pressure while walking. In the MSC, the walkway has a size of 793 cm × 90.0 cm × 0.6 cm. An electronically sensitive area is integrated into the mat including 27 sensor plates (61 cm × 61 cm) with 2,304 sensors per plate (Rowling et al., 2012). The pressure and time courses, the shift of the body’s center of gravity and the rolling behavior of the foot can be analyzed in detail. An increasing gait variability is detected by the evaluation of step length and step time and indicates a beginning gait uncertainty. The GAITRite System is frequently used for clinical as well as research reasons of objective gait assessment and has shown a strong validity and test-retest reliability in various studies. It has proven its clinical importance in the assessment of pwMS (see section “Sensor Floor Plates”) (Bilney et al., 2003; Menz et al., 2004; Sosnoff et al., 2011; Psarakis et al., 2017) and enables the recording of several important gait parameters such as gait speed, stride length and cadence (Bilney et al., 2003).

The Mobility Lab System (APDM, Portland, OR, United States), is a system of portable, wireless network sensors with integrated triaxial accelerometers and gyroscopes function (Fang et al., 2018; APDM Wearable Technologies., 2020). Additional plugins enable the performance of stance assessments and a series of gait and pressure assessments. It provides a valid system for the acquisition of gait data in clinical and research settings (Washabaugh et al., 2017; Morris et al., 2019). In a direct comparison of the Mobility Lab with the GAITRite system, Schmitz-Hübsch et al. have demonstrated that the portable sensors meet the criteria for validity, reliability and objectivity (Schmitz-Hübsch et al., 2016). A recent work has confirmed that the opal sensors are reliable in test-retest analysis and suitable for clinical use (Angelini et al., 2020). We use six portable sensors for the analysis of balance carrying out the Romberg test and for the detailed analysis of different spatial and temporal gait parameters during the 2-MWT.

**FIGURE 2:**
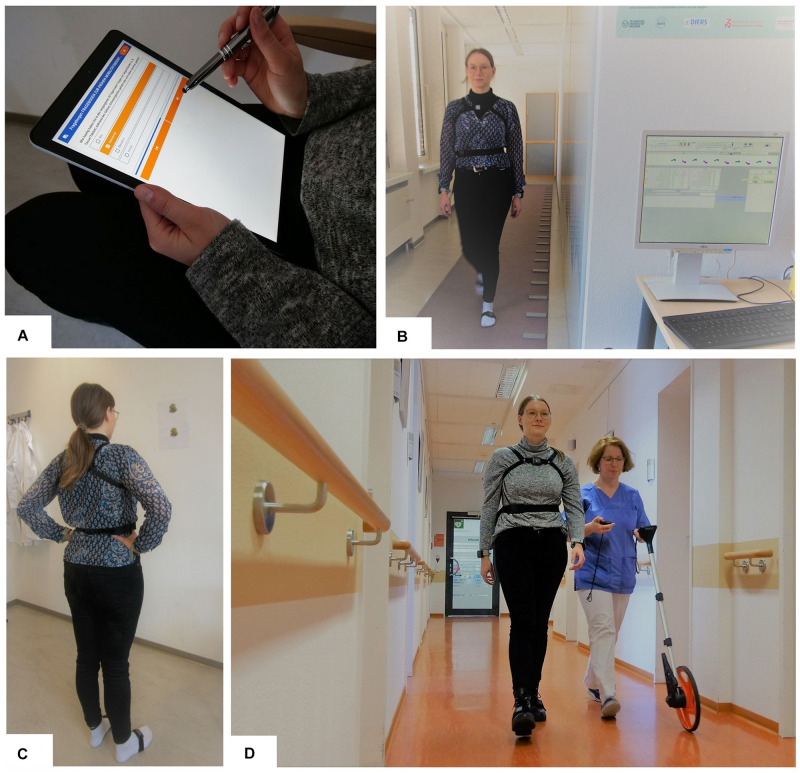
Dresden Protocol for Multidimensional Walking Assessment; **(A)** tablet-based questionnaires; **(B)** normal walking and dual task walking on the GAITRite walking mat; **(C)** balance test with open and closed eyes using the Mobility Lab System; **(D)** Performance of the 2-min walk test with an odometer and motion sensors.

As an extension to the Multiple Sclerosis Documentation System (MSDS^3*D*^), we use two tabled-based questionnaires to measure patient-reported outcomes (PROs): the MSWS-12 and the EMIQ (Hobart et al., 2003; Ziemssen et al., 2016c; Haase et al., 2018; Voigt et al., 2020). Thereby, we also gather the patients’ perspective regarding health outcomes and personal disease status (Figure 2A).

## Test Battery for the Dresden Multidimensional Walking Assessment (DMWA) in Clinical Practice

In the following, we present the assessment tools used in the DMWA. These instruments aim to describe mobility in the domains of walking speed, endurance, quality, balance and self-perception with associated parameters. Overall, the assessments have proven to be suitable for monitoring gait in pwMS and for addressing the lack in reliability and sensitivity to changes observed in the EDSS (Table 2).

**TABLE 2:** Overview of the assessments.

Assessment	Examiner/device	Domains	Parameters*	Validity/Reliability (reference)	Pubmed entries (total/in MS)^†^
EDSS	Physician	Neurological status	Score (1–10)	First publication (Amato et al., 1987) review (Meyer-Moock et al., 2014)	4147/3933
T25FW	MS-nurse/stop watch	Max. walking speed	Time (s)	First publication (in pwMS) (Schwid et al., 1997) review (Kieseier and Pozzilli, 2012; Motl et al., 2017)	387/354
GAITRite a) self-selected speed, single task b) self-selected speed, dual task	MS-nurse/pressure sensors	Quality of gait pattern	Gait speed (cm/s), step length difference (cm), step time difference (s), base of support (cm) functional ambulation profile (%)	First publication (in pwMS) (Bilney et al., 2003; Givon et al., 2009) review (Petraglia et al., 2019)	577/45
Mobility Lab System a) self-selected speed, single task b) self-selected speed, dual task c)2-MWT d)Romberg stand	MS-nurse/motion sensors	a–c) quality of gait pattern d) balance	a–c) gait speed (m/sec) double support (%GCT) stance (%GCT) d) sway area (m^2^/s^4^) sway jerk (m^2^/s^5^)	first publication (in pwMS) (Angelini et al., 2020) review (inertial sensors in general) (Petraglia et al., 2019)	46/1
2-MWT	MS-nurse/odometer wheel	Endurance	Distance traveled (m)	First publication (in pwMS) (Gijbels et al., 2010) review (Kieseier and Pozzilli, 2012)	1021/64
Patient-Reported Outcomes a) MSWS-12 b) EMIQ	Patient/tablet	Mobility self-assessment	Score (0–100) (%)	First publication (MSWS-12) (Hobart et al., 2003) first publication (EMIQ) (Ziemssen et al., 2016c) review (D’Amico et al., 2019) (PRO for MS)	174/173 (MSWS-12) 1/1 (EMIQ)

*2-MWT, 2-Minute Walk Test; T25FW, Timed 25-Foot Walk; EMIQ, Early Mobility Impairment Questionnaire; MSWS-12, 12-Item Multiple Sclerosis Walking Scale; EDSS, Expanded Disability Status Scale; pwMS, people with multiple sclerosis; GCT, gait cycle time. ^∗^Selection of key parameters; ^†^as at June 30, 2020.*

### Assessment of Walking Speed

Walking speed has proven to be a robust marker for the detection of a WI (Bethoux et al., 2016; Motl et al., 2017). For testing walking speed in MS, the best known and currently most widely used test is the T25FW. It is considered one of the most sensitive and reproducible instruments with good validity for quantifying walking ability by measuring the time taken to cover 7.62 m (Cohen et al., 2000; Andreopoulou et al., 2018; Sikes et al., 2020). The examiner first measures and marks the distance and stops the time the patient needs to cover the distance twice. The patient is instructed to walk as safely as necessary, but also as quickly as possible. Two clinically significant benchmarks of progression in MS were identified for the T25FW: patients with a T25FW time of ≥6 s (6–7.99 s) are more likely to be unemployed or in limited employment, and at ≥8 s or higher, social and ambulatory disability decreased significantly (Goldman et al., 2013; Benedict et al., 2016). In the course of monitoring pwMS, a deterioration of more than 20% compared to the previous test is considered a clinically significant difference (Kragt et al., 2006). Revising this finding, Learmonth et al. have identified MDCs for the T25FW in relation to impairment status (Learmonth et al., 2013). For pwMS without an assistive device, 16% deterioration of walk time could be considered significant, whereas for pwMS with assistive device the critical change was higher (34%). With respect to the EDSS, the MDC of pwMS and EDSS <4 was estimated at 12% deterioration, whereas the critical change for pwMS and an EDSS between 4 and 6.5 amounts 36% worsening (Learmonth et al., 2013).

It is recommended to combine the T25FW with sensor-based analysis systems (e.g., Mobility Lab in our test battery). A recent study by Flachenecker et al. reported that significant differences in stride length, walking speed, toe angle, stand- and swing time were more pronounced between pwMS with lower (EDSS ≤ 3. 5) and higher (EDSS 4.0–7.0) disability during testing at fast walking speed (such as the T25FW) (Flachenecker et al., 2019).

### Assessment of Walking Quality With and Without Cognitive-Motor Dual Task

To increase the reliability of the measurements with GAITRite, the patient walks over the mat four times (Figure 2B). The first two walks are performed at a self-selected comfortable walking speed; the following two walks are performed under a dual task challenge, which usually involves two tasks (a word fluency and a calculation task). This method increases sensitivity to show gait abnormalities which cannot be detected during normal walking (Fritz et al., 2019). Since cognitive deficits, for example concentration problems, are a frequent symptom in pwMS, patients with increased cognitive impairment need more attention while walking in order to compensate further disorders (reduced muscle capacity, balance or vision problems). This has been shown in a reduced stride length and walking speed as well as increasing gait variability under dual task conditions (Bridenbaugh and Gschwind, 2011). The fact that subjects are no longer able to hold a normal conversation while walking at normal speed already indicates a significantly increased risk of falling (Lundin-Olsson et al., 1997). Consequently, a study found a significant correlation between risk of fall and walking velocity during dual task (Wajda et al., 2013). Tests combining walking and cognitive task are suggested to better reflect real-life gait impairment in pwMS than a single task setting by simulating a situation closer to real mobility (Leone et al., 2015).

GAITRite parameters have shown to be sensitive in minimally disabled pwMS (Sosnoff et al., 2012). Changes in heel-to-toe progression in pwMS, measured with GAITRite, can distinguish between affected and non-affected limbs of pwMS (Psarakis et al., 2017). The Functional Ambulation Profile (FAP) score (scale 0–100) is used for the overall assessment of walking ability applying the GAITRite technology. It represents the linear relationship of the ratio of stride length and leg length to stride time (Rowling et al., 2012). This FAP score provides an objective analysis of WI and supports the assessment of progression and the effectiveness of treatment approaches. To evaluate its meaningfulness, the FAP score was subjected to a series of tests. The score correlated highly with neurological disability (EDSS, ρ = −0.81) and walking speed (T25FW, ρ = −0.82). Consequently, the FAP score can represent walking impairment in pwMS (Sosnoff et al., 2011).

### Assessment of Balance

**FIGURE 3:**
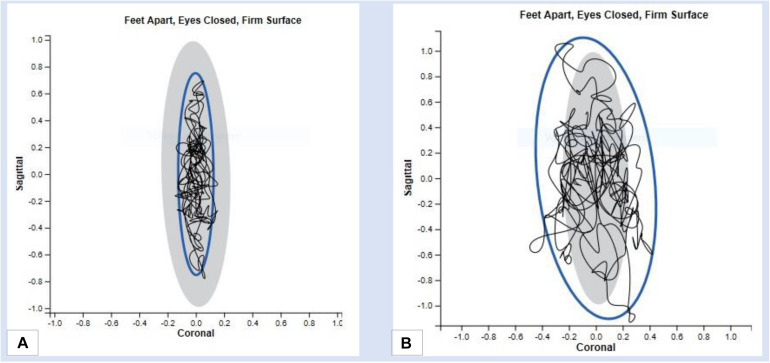
Results of a balance test with the Mobility Lab System **(A)** Fluctuation range in mediolateral and anterior-posterior direction in case of normative reports **(B)** Range of fluctuation in mediolateral and anterior-posterior direction in case of abnormal results.

For balance testing, the Romberg test is used to evaluate variations of the body while standing on a flat surface with the feet hip-wide and parallel to each other (Inojosa et al., 2020b). For comparing the stability, the test is performed with open and closed eyes (Inojosa et al., 2020c). The measuring device (Mobility Lab, APDM) analyses the range of sway from the patient’s center of gravity in the sagittal and frontal plane and it records the anterior-posterior and mediolateral trunk fluctuation range (Gera et al., 2020). The parameters recorded by the measuring device are the fluctuation area and the rate of fluctuation (Figure 2C). A loss of balance during the test phase with eyes closed indicates a positive Romberg test and may be indicative of sensory ataxia (Figures 3A,B).

The system is validated by several studies to provide accurate and reliable measurements of spatial and temporal gait parameters (Washabaugh et al., 2017). Spain et al. (2012) analyzed differences in mobility in pwMS compared to healthy controls with a system of wearable sensors similar to Mobility Lab System. Additionally, they have also successfully used these accelerometers to assess the stability of pwMS in free standing (Spain et al., 2012) showing an increased amplitude of sway in pwMS with eyes-closed condition, while there was no significant difference in walking speed compared to controls. It is known that there are significant changes in the balance of pwMS who are already minimally impaired and it is therefore useful to take the range of trunk fluctuations into account in the mobility analysis (Martin et al., 2006; Pau et al., 2017).

### Assessment of Walking Endurance

PwMS also show limited walking endurance if challenged with longer walking distances. The 6-MWT is the gold standard in many disciplines to test the walking endurance (Goldman et al., 2008). The 6-MWT evaluates the functional capacity but can be considered stressful for pwMS, especially for people with a higher degree of disability. The 2-MWT as a shorter alternative measure, has shown good construct and discriminant validity in pwMS and offers an efficient and practical alternative to the 6-MWT (Scalzitti et al., 2018). In the 2-MWT, patients have to walk continuously for two minutes at a self-selected pace without running. It corresponds to the 6-MWT with the reached distance describing the physical performance. The 2-MWT has shown good validity and reliability in patients with neurological impairment (Rossier and Wade, 2001).

At the MSC Dresden, a modified 2-MWT is performed. In distinction to the standard instruction, where the patient is asked to walk as fast as possible (Bohannon, 2017), our study assistant asks the patient to walk at a normal, self-selected walking speed. The distance is measured with an odometer wheel and additional gait parameters are recorded with the Mobility Lab System. Patients may also use walking aids but they must walk independently. The test is performed in a corridor of about 40m length at our floor in our outpatient clinic (Figure 2D). Where the classical 2-MWT is considered to be representative of the ability to walk, our modified 2-MWT again focuses on analyzing the self-selected walking speed and the resulting walking distance (Gijbels et al., 2011).

### Assessment of Walking-Related Patient-Reported Outcomes

Quality of life is an important factor in assessing progression of the disease and effects of treatments. The benefit of a therapy can best be evaluated by the patient himself, directly from the patient’s perspective (Solari et al., 2005). Therefore, it is important to consider patient’s self-assessment as a relevant outcome in MS assessment of therapy and disability progression (Hobart et al., 2003). The following PRO measures are used at the MSC Dresden:

### Multiple Sclerosis Walking Scale (MSWS-12)

The MSWS-12 is a reliable and robust method to determine walking ability in pwMS, consisting of twelve items (Hobart et al., 2003). Five possible answers are given for each item, which add up to a total value, whereby higher values indicate a more negative influence of WI. Hobart et al. showed that the MSWS-12 is more sensitive than other scales that measure gait ability (Hobart et al., 2003). As a patient-reported outcome measure, the questionnaire is sensitive to assess an increasing disability in walking abilities of pwMS (McGuigan and Hutchinson, 2004). Goldman et al. found that there is a significant change in disability and daily life independence between patients with MSWS-12 scores between 50.0 and 74.9% compared to those who have lower score rates. The latter are more likely to be employed away from home (53.1 vs. 24.2%) and are more often in a full-time employment (94.1 vs. 50.0%). Whereas in the group of MSWS-12 scores from 25.0–49.9% only 15.6% need government healthcare assistance, this number more than triples to 51.5% in the MSWS-12 score group 50.0–74.9%. Having a MSWS-12 score of 75% and above, again, the loss of independency and degree of disability notably rise against compared to the group with lower scores (Goldman et al., 2017). In a survey of 82 pwMS with EDSS scores from 0-6.5, the MWSW-12 showed an MDC of 81/38% (EDSS < 4/4–6.5). Similarly, the MDC was 73% in pwMS without a walking aid and 27% in pwMS that used a walking aid, which also indicates a better responsiveness to changes at higher levels of disability (Learmonth et al., 2013).

### Early Mobility Impairment Questionnaire (EMIQ)

The EMIQ was designed to detect early WI in pwMS. Ziemssen et al. developed an initial 20-item questionnaire, which was modified into a 15-item, and finally a 9-item questionnaire based on exploratory factor analysis (EFA) and item response theory (IRT) (Ziemssen et al., 2016c). In a multi-center, prospective observational study, the psychometric performance of the EMIQ has been evaluated in pwMS with an EDSS of 2.0–6.0. It was demonstrated that the EMIQ is a robust tool to detect mobility disorders in pwMS in clinical practice. In contrast to the MSWS-12, it includes more high-level motor activities and the correlation with cognitive tasks. Initial analyses suggest that the EMIQ has the potential as a screening tool to detect motor deficits in patients at an early stage of MS (Ziemssen et al., 2016c).

### Analysis and Interpretation of the Data

Through the DMWA, we intend to collect data of a large cohort of patients with a confirmed MS diagnosis over a long period in several domains of gait function. A characterization of patients according to demographic characteristics, disease duration, clinical disease course, disease modifying therapy and symptomatic therapies will be performed.

Currently, we generate a set of normative gait data from healthy reference population matched for age and gender to compare baseline results. When the patient is assessed with the DMWA for the first time, these normative data serve as a reference to describe the individual gait pattern and impairments. In the further course of DMWA, we focus on the changes of test results with regard to MDCs (see Chapter 4.2). The results of the multimodal gait analysis are interpreted in cooperation with the examiner, physician and patient.

During the medical consultation with the treating physician, several results are taken into account to get an individual disease profile of the current state, including those markers of disease control or activity, such as MRI results, clinical progression and relapses, lab results (e.g., lymphocyte count, neurofilament light chain) and results of regularly assessments of the DMWA, the EDSS and the Multiple Sclerosis Performance Test (Rudick et al., 2014). For results indicating recurrent or persistent disease activity or further clinical progression, the obvious aim is to optimize current treatment through escalation of immunomodulating therapies and symptomatic treatment. Additionally, we discuss supportive approaches like rehabilitation programs or physical therapy, logopedics and ergotherapy to reach a best possible disease outcome. However, due to highly variable interindividual clinical presentation and growing amount of defining biomarkers and surrogate endpoints, an efficient MS management requires the personalization of each disease presentation, which favors our objective of a tailored treatment approach (Ziemssen et al., 2016b, 2017; Noffs et al., 2018). Our innovative technologies like the GAITRite and Mobility Lab System allow straightforward data acquisition. However, one of the main challenges is to link these data to clinical meaningful outcomes in order to improve patient- and therapy management and to achieve the best possible treatment outcome. It leads to the question of how to analyze big data and integrate results into existing clinical evidence. For instance, we have used an artificial intelligence approach for data interpretation to achieve a structured data analysis for our patients (Trentzsch et al., 2019; Weidemann et al., 2019b).

## The Vision for a Holistic Walking Assessment in MS

Our vision is to combine future clinical evaluation of walking with the monitoring of gait in real life beyond our center. In this way, a comprehensive patient assessment would be possible not only during clinical visits, but also in the daily life of pwMS through data collection via the usage of smart devices like smartphones or body worn motion sensors. In a first step, we chose two systems that can be used in mobility monitoring at home: the GENEActiv Original (Activinsigths Ltd., United Kingdom) and socks with integrated sensory insoles. The GENEActiv device is a body worn intertial sensor, which measures acceleration via micro electromechanical system sensors. It is waterproof and therefore can be worn 24h and contains a temperature sensor to detect wear time. As a device developed for research purposes, the GENEActiv can be compared to ActiGraph products (GENEActiv, 2019). First validation studies have been promising, but to the authors knowledge, the device has not been used in pwMS yet (Pavey et al., 2016). The sensory socks operate with force sensing resistor sensors, integrated in an insole (IEE S.A., Luxembourg). The insole allows for force analyses with regard to the area of the foot where the force is applied (IEE, 2020). We seek to examine those devices in future projects of mobility assessment at our center in order to evaluate their suitability for assessment in daily living.

Especially through contributing real-life data, pwMS would play a more active and responsible role in their own monitoring of progression. Finally, this may increase compliance and optimize disease outcomes in the long run.

## Prospects for the Future

The DMWA could become an important step to quantify walking limitations in a comprehensive way at a low threshold. Although the EDSS is the most frequently used tool for assessing disability in pwMS, it has significant limitations in terms of reliability and sensitivity (Dandu et al., 2018). Therefore, we go one step further with the DMWA to reach our goal of obtaining a sensitive and straightforward quantification of walking impairment.

Currently, there are already similar protocols in place to detect changes in walking function in pwMS (Decavel and Sagawa, 2019). However, the combination of standard tests and new sensor-based methodology increases sensitivity and reliability. The sensor-based gait analysis objectively supports the clinical evaluation of the limited parameters of standard walking assessment. In addition, the generated data can be analyzed by innovative algorithms using machine-based learning.

As an innovative technology, walking assessment may play a more important role in the future. Digital monitoring at home is a promising approach that needs to be evaluated in further studies. There are already alternatives without body sensors, so-called ambient system devices for use at home, such as the new Echo5D technology. First results have demonstrated that a reliable and continuous assessment at home is possible applying this infrared technology (Smith et al., 2018).

**FIGURE 4:**
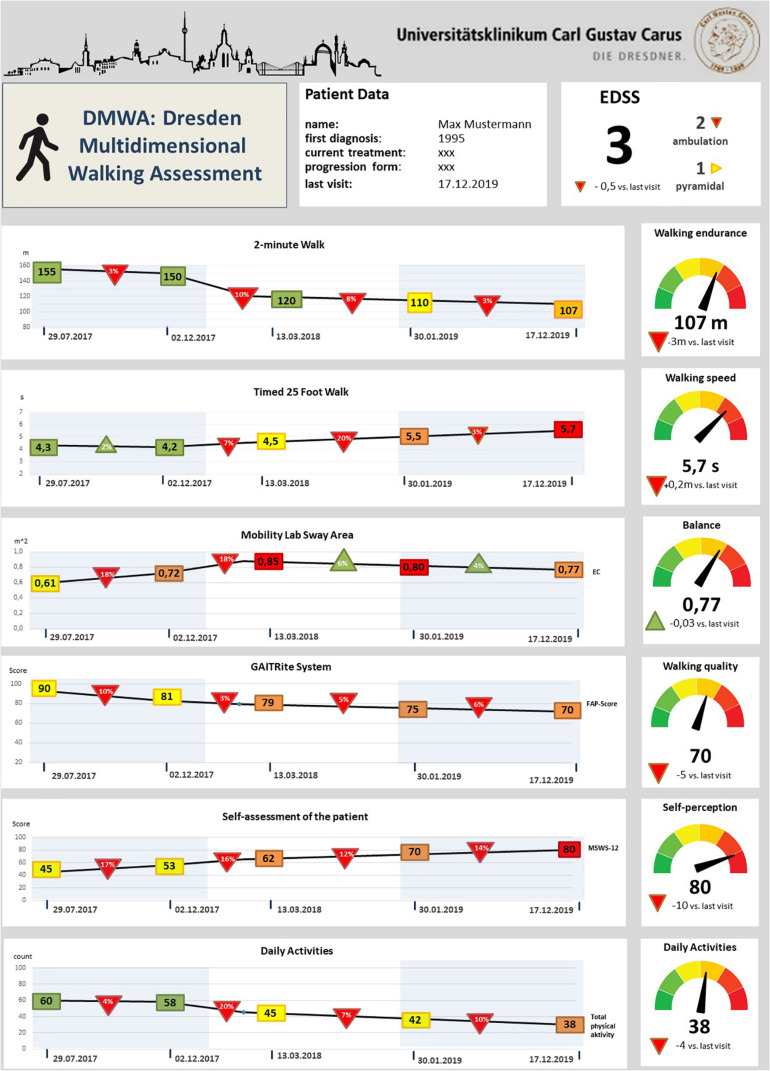
Draft of the future vision of a dashboard for multidimensional gait analysis in multiple sclerosis. Rectangles indicate the respective assessment value at the time point of the routine visit; colors point out the relationship with the reference values (green represents within norm, yellow small and red high deviations from the reference values for each variable). Triangles show the relative longitudinal alterations of the values with the last visits – red triangles indicate a worsening and green triangles an improvement compared to the last routine check; In the right column the absolute values of the current gait analysis evaluation are illustrated, the traffic light system represents the patient’s performance according to the reference values; EC, Eyes closed; MSWS-12, 12-point gait scale Multiple Sclerosis; EDSS, Extended Disability Status Scale; FAP-Score, Score of the functional ambulation profile.

In the future, we aim to design a dashboard for walking assessment in MS as part of our multidimensional digital patient management system MSDS^3*D*^ (Haase et al., 2018; Ziemssen et al., 2020), showing the results of our clinical multidimensional walking assessment and daily smart monitoring longitudinally. This DMWA dashboard uses a kind of traffic light system to indicate the domains in which pwMS present abnormal outcomes. Furthermore, the follow up and development of walking function over time are visualized (Figure 4). Such a way of presentation of complex data may not only facilitate clinical decision making and monitoring of progression but also may support disease awareness of pwMS and enable informed discussions about next therapeutic steps based on the results in the walking assessment dashboard.

As a limitation, results from controlled laboratory conditions at a given time do not capture the influence of environmental conditions or daily variations. In future, more attention has to be given to this aspect and mobility data should be recorded continuously in the real-life environment. However, innovative technological approaches already exist for the monitoring of gait and balance disorders in everyday life (Dandu et al., 2018; Weidemann et al., 2019a). The use of the GAITRite system, the Mobility Lab and the collection of walking endurance require the availability of equipment and extended facilities. If these are not available, we recommend at least a minimum set consisting of the core elements T25FW, MSWS-12 and EMIQ, which can also be understood as a screening approach.

## Conclusion

Walking impairment has a significant impact on the life and quality of life of pwMS being often bothered with many additional MS-associated symptoms. Consequently, pwMS should be given high diagnostic priority in order to monitor the walking ability and to detect subtle disease changes over time. A comprehensive multidimensional walking assessment is crucial to monitor disease activity and phenotype individual characteristics in a multifocal disease such as MS (Ziemssen, 2019). Therefore, a straightforward but also detailed quantitative analysis of gait is an important part of the individual monitoring. Our DMWA has implemented new methodological concepts besides standard elements to apply research findings in the day-to-day clinical practice. The protocol presented here has been implemented at the Dresden MS Center since 2018. In a future work, we will present first data showing our experience with the implementation of DMWA in clinical practice.

## Data Availability Statement

The original contributions presented in the study are included in the article/supplementary material, further inquiries can be directed to the corresponding author.

## Ethics Statement

Written informed consent was obtained from the individual(s) for the publication of any potentially identifiable images or data included in this article.

## Author Contributions

KT and MW contributed to, conception and writing of manuscript. CT created the tables and figures. DS, KA, MS, and RH reviewed the manuscript for intellectual content. TZ contributed to concepting, and reviewing the manuscript for intellectual content. All authors contributed to the article and approved the submitted version.

## Conflict of Interest

The authors declare that the research was conducted in the absence of any commercial or financial relationships that could be construed as a potential conflict of interest.
